# Drug-tolerant persister cells: the deadly survivors in hematological malignancies

**DOI:** 10.3389/fphar.2026.1740281

**Published:** 2026-04-14

**Authors:** Qianying Ma, Tingyong Cao, Lanxia Zhou, Juan Cheng, Li Zhao

**Affiliations:** 1 The First Clinical Medical College, Lanzhou University, Lanzhou, China; 2 Department of Hematology, The Second Hospital & Clinical Medical School, Lanzhou University, Lanzhou, China; 3 Gansu Provincial Clinical Medical Research Center for Molecular Diagnosis and Treatment of Hematological Diseases, The First Hospital of Lanzhou University, Lanzhou, China; 4 The Laboratory Center, The First Hospital of Lanzhou University, Lanzhou, China; 5 Department of Hematology, The First Hospital of Lanzhou University, Lanzhou, China

**Keywords:** drug resistance, drug-tolerant persister cells, hematological malignancies, minimal residual diseases, reversible

## Abstract

Although the prognosis of patients with hematologic cancers has improved significantly owing to the advance of radiotherapy, chemotherapy, targeted therapies, immunotherapies, and hematopoietic stem cell transplantation therapy, disease relapse and refractory are still very prevalent and remain the major obstacles for long-term patient survival. Recent evidence suggests that drug-tolerant persister (DTP) cells are considered to be the cellular reservoir for tumor relapses and drug resistance formation. DTP cells refer to tumor cells that are acquired tolerance to anticancer drugs during exposure. Distinct from genetic mutations which cause stable drug-resistance, drug-tolerance in DTP cells is reversible which is primarily mediated by non-genetic alterations including changes in epigenetic modification, metabolic reprogramming, and/or transcriptional regulation. However, under prolonged or repeat drug-exposure, DTP cells acquire mutations in genes that are required for drug-treatment response and become stable drug-resistance. Targeting stable drug-resistant cell therapy is very challenge which needs novel drugs. Thus, targeted DTP cells therapy is particularly interesting which only needs to inhibit the essential adaptive pathways to prevent DTP cells formation or restore drug-sensitivity to DTP cells. During the past 15 years, significant efforts have been made to understand the mechanisms that drive the generation of DTP cells and define unique vulnerabilities that can be exploited for targeting DTP cells therapies. Combination of drug with adaptive pathway inhibitors has been attempted to prevent recurrence and refractory for durable treatment efficacy. Here, we review the advances of DTP cells research in hematologic cancers and summarize the phenotypic and functional characterizations of DTP cells defined in different research models. By deliberating the mechanisms of DTP cells generation and evolution, we discuss the potential therapeutic strategies of targeting DTP cells for more an effective treatment of hematological malignancies.

## 1 Introduction

Hematologic malignancies are a group of heterogeneous cancers including leukemia, lymphoma and multiple myeloma, that occur in the blood, bone marrow, and/or lymph nodes characterized by uncontrolled abnormal growth of hematopoietic cells and lymphocytes at different stages of differentiation (Auberger et al., 2020; Uribe-Herranz et al., 2021). In recent years, owing to the advances of radiotherapy, chemotherapy, targeted therapies, immunotherapies, and hematopoietic stem cell transplantation therapy, the prognosis for patients with hematologic tumors has been significantly improved. Currently, drug-resistance and disease relapses are the major obstacles for the cure of hematologic malignancies. Thus, the long-term survival of patients remains a significant challenge (Auberger et al., 2020; Hofmann et al., 2023). Indeed, whatever the kind of treatment, malignant hematopoietic cells consistently develop cellular strategies to adapt to and survive from therapeutic drugs. Such adaptations may involve different molecular and cellular mechanisms, including the acquisition of mutations, epigenetic modifications, the regulation of Tumor microenvironment and cell-in-cell (CIC) phenomenon (Auberger et al., 2020; Choe et al., 2022; Giannotta et al., 2023). Clinical studies demonstrate that a small percentage of patients show no response to current treatments due to the primary resistance of their cancer cells. Novel medications are urgently needed for such patients to effectively kill the drug-resistant cells. For most responders, due to the failure of complete elimination of tumor cells, recurrence of tumors from the minimal residual diseases (MRD) are ultimately inevitable (Auberger et al., 2020; Hofmann et al., 2023; Calderon et al., 2024). To further improve the long-term survival of this group of patients, novel therapeutic strategies are needed to target MRD for preventing disease relapse.

MRD is developed from a small subset of cancer cells that survive during treatments owing to either primary or acquired tolerance/resistance to anti-cancer drugs (Holohan et al., 2013; Gong et al., 2025; Chou et al., 2025). Diverse mechanisms have been defined to explain how MRD is developed in patients (Chou et al., 2025). In some patients, MRD develops from treatment selection of drug-resistant subclones. Drug-resistance in such patients is irreversible due to mutations of genes that are required for drug-response, novel drugs are needed to treat such patients to overcome the drug-resistance (Ding et al., 2012; Tyner et al., 2018). However, in majority patients, MRD is developed from cancer cells without genetic mutations. The term of drug-tolerant persister (DTP) cells has been employed to explain the non-genetic alterations-associated drug-tolerance. DTP cells are a subpopulation of cancer cells with a reversible “drug-tolerant” state that can survive drug therapy through non-genetic mechanisms. A growing body of evidence suggests that DTP cells are the key cellular reservoir for cancer relapse and the major non-genetic barrier for effective cancer treatment (Sharma et al., 2010; Marine et al., 2020; Kalkavan et al., 2022; Pu et al., 2023; He et al., 2024; Li et al., 2025). In addition, DTP cells can survive long enough during continuous drug treatment and progress to stable drug-resistant cells due to genetic evolution (Pu et al., 2023; He et al., 2024; Li et al., 2025). Currently, our ability to target cancer cells with stable drug-resistance is limited. However, unlike the stable drug-resistant cancer cells, drug-sensitivity in DTP cells can be effectively reversed after drug treatment is discontinued. Most importantly, such reversable process can be induced by medications through targeting vulnerable transcriptional, metabolic and/or epigenetic pathways (Vinogradova et al., 2016; Echeverria et al., 2019; Kurppa et al., 2020; Pu et al., 2023; He et al., 2024). Therefore, one of attractive therapeutic strategies for cancer treatments is to eliminate DTP cells during the initial treatments or stop the evolution of drug resistance at the early stage of DTP cells development.

During the past 15 years, DTP cells have been studied in many types of human cancers including breast cancer, prostate cancer, melanoma, gastric cancer, and colorectal cancer (Shen et al., 2020; Rehman et al., 2021; Chang et al., 2022; Wang et al., 2024; Xu et al., 2026). However, to overcome disease relapse in hematopoietic malignancies, most previous studies focused on cancer stem cells (CSCs) (Shlush et al., 2017), the roles of DTP cells in disease relapse and refractory are only realized recently. Here we summarize the current research advances of DTP cells in hematopoietic malignancies and discuss the potential molecular mechanisms by which DTP cells generate in different hematopoietic cancers. By elucidating the essential stress mitigation pathways that facilitate the survival of DTP cells during treatment, we discuss the potential treatment strategies to target DTP cells for durable cure of hematopoietic malignancies.

## 2 Overview of DTP cells

### 2.1 The concept of DTP cells

The fundamentals of DTP cells were first described in the 1940s to depict the phenomenon of antibiotic treatment-induced non-genetic tolerance in a small subset of bacteria (Russo et al., 2024). Such a concept was introduced to cancer treatment in 2010 to explain the reversible drug-tolerant state of cancer cells to anti-cancer therapies. It was found that many cancer patients who respond well to anticancer treatments and reach partial or complete remissions (PR or CR) with MRD during initial therapy; however, majority of them experience disease relapses during treatment intervention (medication-holiday). Fortunately, the MRDs in most cases are derived from cancer cells with a reversible drug-tolerant state, meaning that relapsed disease in such patients remains responsive to the same treatments (Cara and Tannock, 2001; Kurata et al., 2004; Yano et al., 2005). In 2010, Sharma et al., (Sharma et al., 2010), first described DTP cells in study of lung cancers and found that a small subset of cancer cells acquire a reversible drug-tolerate phenotype during EGFR inhibitor or cisplatin treatments. After that, DTP cells have been identified in a wide range of cancers in response to both chemotherapy and targeted agents including breast cancer (Chang et al., 2022), colorectal cancer (Rehman et al., 2021), prostate cancer (Wang et al., 2024), glioblastoma (Liau et al., 2017), melanoma (Shen et al., 2020), ovarian cancer (Böpple et al., 2024) and hematopoietic malignancies (Ge et al., 2021a; Morgenstern et al., 2025).

### 2.2 The origins of DTP cells

**FIGURE 1:**
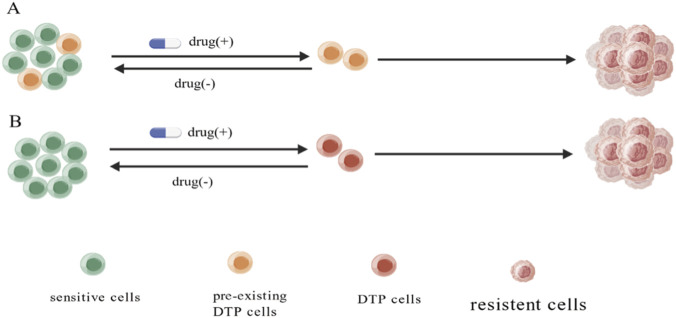
Origins of DTP cells. Selection and induction, two mechanisms have been proposed to explain the generation of DTP cells. **(A)** In selection mechanism, a small subset of cancer cells having DTP cells features which are preexisted in treatment-naïve cancer tissues. Upon treatment, drugs selectively eliminate the sensitive cells which result in DTP cells development from accumulation of pre-existing persisters. **(B)** In induction mechanism, DTP cells are generated by drug-induced toleration in treatment-naïve cancer cells in a stochastic manner. In both cases, prolonged or repeated drug-exposure will induce genetic mutations and lead to stable drug-resistance. (Created with BioGDP.com).

Clonal selection of pre-existing drug-tolerant cells and drug-induced acquisition of drug-tolerance, two mechanisms have been proposed to explicate the origins of DTP cells (He et al., 2024) (Figure 1). Although these two mechanisms were reported by different studies which were most likely drug and cancer specific, they might not happen exclusively. In certain cancer models, both mechanisms might involve. In the clonal selection mechanism, it was found that a subpopulation of cells with drug-tolerance/resistance can be detected in patient cancer tissues prior to treatments. Such cells exhibit certain unique biological features (such as stemness, slow cell cycle/quiescence, multidrug-resistance and/or epigenetic/metabolic alterations) that are inherently tolerated to the treatments (partially drug-resistant) (Miranda Furtado et al., 2019; Francescangeli et al., 2020; Xu et al., 2022; Raha et al., 2014). In drug-induction mechanism, it was suggested that all cancer cells have the equivalent capacity to become DTP cells (Rehman et al., 2021). DTP cells are induced stochastically upon drug treatment by inducing a drug-tolerate state in a small fraction of drug-sensitive cancer cells through promoting transcriptional, epigenetic and/or metabolic adaption (Echeverria et al., 2019; Vinogradova et al., 2016; Kurppa et al., 2020; Pu et al., 2023; He et al., 2024). Because the drug tolerance of DTP cells is reversible, diseases that are relapsed from DTP cells still respond to the treatment with original drug. However, under persistent drug-exposure, DTP cells will become stable drug-resistance due to the acquiesce of genetic mutations.

### 2.3 Biological characteristics of DTP cells

Diverse biological characteristics have been reported to describe DTP cells in different studies (Kalkavan et al., 2022; He et al., 2024), including 1) cell cycle: slow-cycling or quiescent state (G0/G1 and G2-M phase of the cell cycle) (Zhang et al., 2022; Haderk et al., 2024; Morgenstern et al., 2025); 2) phenotype: stem-like (Hangauer et al., 2017; He et al., 2024), senescence-like (Kurppa et al., 2020) or embryonic diapause-like (Rehman et al., 2021; Russo et al., 2024); 3) metabolism: increased oxidative phosphorylation (OXPHOS) and/or fatty acid oxidation (FAO) (Farge et al., 2017); 4) stress response: elevated oxidative stress (Raha et al., 2014); 5) morphology: activation of the epithelial to mesenchymal transition (EMT) program (Hangauer et al., 2017; Böpple et al., 2024); 6) immune response (Russo et al., 2024); 7) plasticity: capable of transitioning between different cellular states (Risom et al., 2018). DTP cells from different cancer models only present some of these biological characteristics. Importantly, all these biological characteristics can be reversed following treatment cessation (Sharma et al., 2010; He et al., 2024). In addition, DTP cells may develop epigenetic memory which display increased persistence upon drug rechallenge, consequently escalating the frequency of DTP cells formation and potentially enhancing rates of drug resistance (Russo et al., 2024; He et al., 2024). These biological features are not mutually exclusive and often coexist within DTP cells, regulated by common upstream mechanisms such as epigenetic modification, transcriptional regulations, metabolic remodeling, and tumor microenvironment (TME) (He et al., 2024). However, the biological characteristics of DTP cells can be significantly influenced by not only treatment agents and dosages, but also the complex interplay between clonal dynamics and tissue-specific factors, as well as interactions between DTP cells and the TME (Russo et al., 2024). In addition, DTP cells in most cancer tissues represent a dynamic state of tumor cell adaptation to drug stress (Russo et al., 2024), thus, even within a single tumor, distinct phenotypic DTP cells can be detected. Consequently, DTP cells are phenotypic heterogeneity and challenging to define.

### 2.4 Mechanisms of DTP cells formation and drug resistance

Given these characteristics of DTP cells, increasing attention has been paid to its formation and drug resistance. However, the process of DTP cells formation is still not fully understood and may vary among cancer cells. Specific adaptive non-genetic alterations in transcriptional profile (Arasada et al., 2018; Kurppa et al., 2020), epigenetic landscape (Sharma et al., 2010; Vinogradova et al., 2016; Guler et al., 2017), signaling pathways and/or metabolic regulation (Hangauer et al., 2017; Echeverria et al., 2019; He et al., 2024) have been detected in DTP cells compared to parental cancer cells regardless of whether DTP cells are selected from a pre-existing persisters or emerge during drug exposure. In the DTP cells generated from clonal selection, such adaptive non-genetic alterations have already existed in the pre-existing persisters, whereas in the DTP cells generated from drug-induction, such adaptive non-genetic alterations are dynamically induced by drugs through stepwise reprogramming (He et al., 2024). In both cases, the alterations in epigenetic landscape lead to transcriptional regulation of genes that promote metabolic fitness and mitigate drug-induced toxic stress (Ravindran Menon et al., 2020; Russo et al., 2024; Liu et al., 2025; Singh et al., 2025). These genes are commonly involved in signaling pathways for cell survival, drug resistance, cell cycle, energy metabolism, signaling pathways, OXPHOS, redox regulation and immune evasion (Hangauer et al., 2017; Kurppa et al., 2020; Oren et al., 2021; Li et al., 2023; Russo et al., 2024; Zhang Y. et al., 2025; He et al., 2024). After the cessation of treatment, all transcriptional, epigenetic and/or metabolic alterations can be reversed dynamically to a treatment-sensitive state. As consequence, the relapsed cancers from DTP cells are still response to the drug treatment (He et al., 2024). This is the most critical distinction between DTP cells and drug-resistant cells, DTP cells can re-enter the proliferative state during tumor therapy and subsequently provide a potential cellular reservoir for the emergence of acquired resistance mechanisms in antitumor therapy during continued treatment. Additionally, their characteristics (Table 1), including post-drug withdrawal reactions, survival mechanism, treatment and status of proliferation, vary (Liu et al., 2025). The mechanisms of DTP cells’ formation are not universal to all types of cancers, cancer type and drug specific mechanisms were reported in different model systems, highlighting the complex nature of this phenomenon.

**TABLE 1:** Comparison of tolerant DTP cells and resistant cells.

​	DTP cells	Resistance cell
Characterization	Transient resistance Reversible	Irreversible
Post-drug withdrawal reactions	re-forming drug-sensitive groups	Complete loss of reversibility of the drug-resistant phenotype
Survival mechanism	Non-genetic plasticity	Genetic alterations: acquired mutations
Treatment	Original program effective	Change of treatment program
Status of proliferation	Non/slow proliferation	Continued proliferation

### 2.5 Cancer stem cells and DTP cells

Same as cells in normal tissue, cancer cells in tumor tissue are also heterogeneous, composing cells at different stages of differentiation (Nassar and Blanpain, 2016; Ayob and Ramasamy, 2018). CSCs are a subpopulation of cancer cells with stem cell-like properties, including self-renewal ability and multi-lineage differentiation (Nassar and Blanpain, 2016; Ayob and Ramasamy, 2018). CSCs reside at the apex of the differentiation hierarchy within cancer and can produce cancer progenitors and then cancer blasts to regenerate cancer tissues during relapse and in transplantation models (Singh et al., 2004; Clevers, 2011; Nassar and Blanpain, 2016). The concept of CSCs has been proposed to explain how cancer is initiated at the early stage of cancer development and becomes therapeutic resistant and spreads during disease progress (Nassar and Blanpain, 2016; Makena et al., 2020). Accumulated evidence suggests that compared to cancer progenitors and blasts, CSCs are relatively slow cycling, express high levels of survival and multidrug resistant genes, or exhibit unique epigenetic/metabolic activities (Nassar and Blanpain, 2016; Butti et al., 2019; Huang et al., 2020; Shiokawa et al., 2020). As consequence, CSCs are intrinsically tolerant to most conventional therapies (Makena et al., 2020). Most current therapies can largely kill cancer progenitors and blasts to induce disease remission, however, due to the failure in elimination of CSCs, cancer relapse is inevitable in most patients (Shibata and Hoque, 2019; Walcher et al., 2020; Huang et al., 2020). Cancers that are regenerated from CSCs still maintain the heterogeneous and differentiation hierarchy of their parent cancers and might be still sensitive to conventional therapies (Yang et al., 2024; Huang et al., 2020; Nassar and Blanpain, 2016); however, the frequencies of CSCs might be increased during each relapse and become refractory eventually (Aramini et al., 2022; Masciale et al., 2022). The biological characteristics of CSCs are primarily regulated by epigenetic and metabolic machinery and regulated by tumor microenvironment which can be reversed by targeting venerable vulnerable pathways to induce differentiation (Nassar and Blanpain, 2016; Ayob and Ramasamy, 2018; Huang et al., 2020; Li et al., 2024; Saw et al., 2024). Therefore, targeting CSCs is a key area of cancer research during the past 30 years, with the potential to more effectively prevent relapses for lasting cancer treatments (Makena et al., 2020). However, for DTP cells exhibiting a partial stem cell phenotype, they are slow-proliferating tumor cells induced by external drug stimuli, which can regain drug sensitivity after treatment cessation, but lack the high proliferative activity and self-renewal capacity characteristic of stem cells. In this sense, many cells previously labeled as CSCs can be redefined as DTP cells that exist for survival rather than as stem cells dedicated to differentiation and proliferation (He et al., 2024).

### 2.6 Tumor *microenvironment* (TME) and DTP cells

The TME plays an important role in the development and progression of hematological malignancies (Das et al., 2023). Accumulating evidence demonstrates that the survival and evolution of DTP cells are similarly regulated by dynamic interactions with the TME (Dhanyamraju et al., 2022). For instance, in a TKI-induced lung cancer DTP cells model, TKI treatment was shown to indirectly induce phenotypic alterations in cancer-associated fibroblasts (CAFs), which enhance DTP cells survival *via* upregulation of NF-κB and STAT3 signaling (Moghal et al., 2023). Additionally, TKI-driven macrophage interactions can sustain the DTP cells phenotype through activation of the PROS1-AXL signaling axis (Zhang X. et al., 2025). Similarly, Microenvironmental pressures emerging during cancer evolution foster tumor heterogeneity (TH) and facilitate the emergence of drug-resistant clones (Mancini et al., 2024).

## 3 DTP cells in hematological malignancies

DTP cells have been better studied in a spectral of solid tumors and provide feasibility for new therapeutic targets in solid tumors. The role of DTP cells in the treatments of hematologic tumors has only been explored recently.

### 3.1 DTP cells in Acute myeloid leukemia (AML)

**FIGURE 2:**
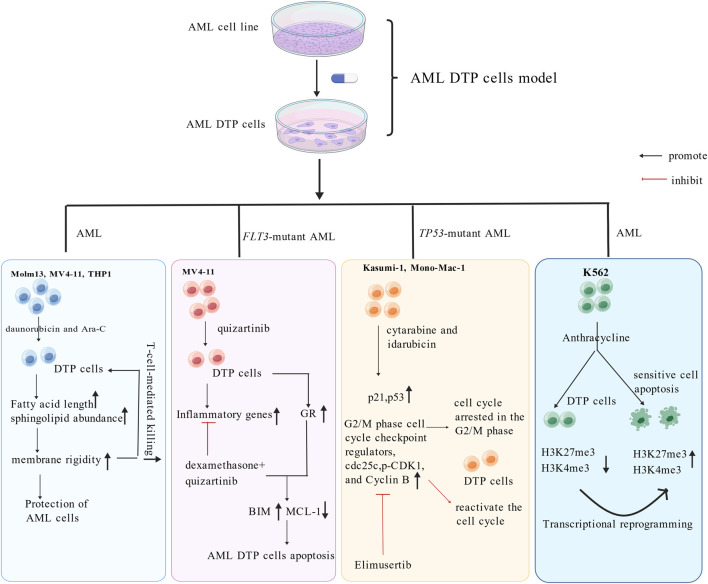
Modeling and Mechanisms of DTP cells in AML diagnosis-relapse paired cases analyzed (Created with BioGDP.com). GR: glucocorticoid receptor; BIM: Bcl-2-interacting mediator of cell death; MCL-1: mantle cell lymphoma-1.

AML is a heterogeneous group of hematologic malignancies and the most common type of acute leukemia characterized by uncontrolled expansion of immature myeloid blasts (Löwenberg and Rowe, 2016; Goldman et al., 2019; DiNardo et al., 2023). AML develops from genetically mutant hematopoietic stem cells (HSCs) (Welch et al., 2012). Despite the development of targeted therapies for AML with certain genetic mutations such as FLT3 or IDH1/2 mutations, current standard care for AML is still intensive chemotherapy followed by HSC transplantation (HSC-T) as salvage treatment (Liu, 2021). Traditionally, leukemia stem cells (LSC) have been thought to play a major role in drug resistance and disease relapse in chemotherapy treated AML patients (Shlush et al., 2017; Stelmach and Trumpp, 2023). These early studies imply that DTP cells in AML might be enriched with LSCs. However, this notion was challenged by several recent studies. Nuno et al. (2024) analyzed of paired diagnosis-relapse samples from cohorts of AML cases and found that 40% of relapses occurred without changes in driver mutations. Single-cell ATAC-seq analysis suggested that epigenetic evolution in non-LSC populations drives the relapses in such cases. In addition, Farge et al. (2017) reported that AraC-resistant AML cells in xenograft models exhibit a reversible high FAO and OXPHOS status without enrichment of LSCs. This study suggested that enhanced mitochondrial FAO-OXPHOS is a key contributor to chemoresistance in AML. Of course, DTP cells are not entirely different from LSCs. van Gils et al. (2022) found that doxorubicin induced anthracycline-DTP cells displaying LSC features without upregulation of histone 3 lysine 27 (H3K27) and H3K4 tri-methylation bivalent state. Subsequently, researchers established drug-induced AML DTP cells models. Morgenstern et al. (2025) reported that daunorubicin and Ara-C combination-induced DTP cells display a transient increase of plasmic membrane rigidity due to the increased fatty acid length elongation and sphingomyelin abundance. FLT3 inhibitor quizartinib induces the upregulation of inflammatory pathways in AML DTP cells and thereby confers susceptibility to anti-inflammatory glucocorticoids (GCs) (Gebru et al., 2020). Kim et al. (2024) reported that in TP53-mutant AML cells, cytarabine and idarubicin combination increase the levels of the p53 and p21 proteins, and upregulated the G2/M-phase cell-cycle checkpoints regulators cdc25c, p-CDK1, and Cyclin b in DTP cells, causing cell cycle arrest in G2/M phase and drug-resistance. Concurrently, Bell et al. (2019) also documented DTP cells resistance resulting from epigenetic reprogramming. Using both single-cell RNAseq and Crispr-function screening assays, researchers found that BET inhibitor induces a Lsd1 (Kdm1a)-mediated epigenetic resistance by driving transcriptional adaptation. Such drug-resistance can be overcome by Lsd1 inactivation which facilitates the binding of Pu.1 and Irf8 to new enhancers that regulate the expression of key survival genes. Figure 2 analyzes the modeling and mechanisms of DTP cells in AML diagnosis-relapse paired cases. All these studies suggested that in many AML patients, DTP cells development is induced by drugs through triggering epigenetic evolution and/or metabolic reprograming which mediate adaption to drug-stress in a subset of AML cells without a clear LSC feature. Such epigenetic and metabolic adaption mitigates the toxic stress of drugs by activating transcriptional programs and signaling pathways.

### 3.2 DTP cells in *acute* lymphoblastic leukemia (ALL)

T-ALL is an aggressive type of malignant tumor of T lymphocytes. Although pediatric cases of T-ALL have a cure rate exceeding 80% with current intensified chemotherapies, the outcome of T-ALL patients with primary resistant or relapsed leukemia remains extremely poor (Winter et al., 2015). Constitutive activation NOTCH1 signaling due to genetic alterations of NOTCH1 or FBXW7 genes is a major oncogenic driver in major of T-ALL patients (Weng et al., 2004; Liu et al., 2017), and the disease progression in these patients can be inhibited by NOTCH1 inactivation by small molecule γ-secretase inhibitor (GSI) (Palomero and Ferrando, 2009). However, due to the emergence of drug resistance and toxicity, clinical efficacy has been suboptimal. Researchers are attempting to address this challenge through epigenetic mechanisms. Knoechel et al. (2014) reported that GSI-induced DTP cells in NOTCH1-dependent human T-ALL cells are derived from rare preexisted persisters which display epigenetic modification-related activation of distinct signaling and transcriptional programs. BRD4 inhibitor JQ1 treatment can reverse such epigenetic modification and sensitize DTP cells to GSI treatment. GSI and JQ1 combination synergistically inhibit T-ALL in patient-derived xenograft model.

B-ALL is the most common pediatric malignancy which also occurs in adults. Currently, 5-year event-free survival rate of B-ALL is about 90% in children. Treatment-related death and leukemia relapse remain the leading causes of mortality (Smeets et al., 2024). Vincristine induces generation of DTP cells in 30 days in stromal co-culture of ICN13 PDX B-ALL cells (MLL-AF4^+^). Using Cas9/CRISPR screening assay, Zhang et al. (2023) identified genes that are required for the generation of vincristine-induced DTP cells including *MYH9* (an ATP-driven motor protein), *NCSTN, KIAA2013, CD44 and ABCC1/MRP1*. This suggests that addition of ABCC1 inhibitor during induction therapy could provide benefit in eradication of MRD in patients treated with a chemotherapy regimen that includes vincristine. Using proteomics and glycoproteomics analysis, Oliveira et al. (2024) showed alterations in a number of glycan metabolic enzymes in vincristine-DTP cells which are associated with significant changes in glycosite-specific modulation of cell surface and lysosomal proteins. Such glycosite-specific modulation of cytoplasm proteins might provide potential neoantigens for development of immunotherapy for targeting DTP cells. In addition, WEE 1 kinase is a crucial protein involved in regulating the G2/M cell cycle checkpoint and is essential for proper cell cycle progress (Moiseeva et al., 2019). B-ALL cells with *MLL*-rearrangements (*MLL-r*) express high levels of WEE 1 and dependent on WEE1 for their survival. Malyukova et al. (2024) found that WEE 1 inhibitor (AZD 1775)-induced DTP cells in human *MLL-r* B-ALL obtain a pre-BCR^+^/BCL6^+^ state with reduced stemness markers due to an elevated chromatin access at BCL6 and pre-BCR gene loci and transcription factor motifs of NF-κB, MEF2D, and the metabolic regulators SREBF and LXR. Such epigenetic reprogram results in a reversible cell state switching which is AZD1775 specific because it is not observed in L-asparaginase, cytarabine, or doxorubicin treatment. Such AZD 1775-induced DTP cells is dependent on pre-BCR signaling and lipid metabolism for survival and proliferation recovery because they are sensitive to the treatment pre-BCR inhibitors (dasatinib or itretinib) and lipid metabolism inhibitors (such as mTOR inhibitor AZD2014 and SREBP inhibitor fatostatin).

*MLL-AF4* is a type *Mll-r* which is oncogenic driver detected in >80% of infant B-ALL cases (Pieters, 2009). *MLL-AF4* B-ALL is highly aggressive and has a very poor overall prognosis which tends to be refractory to conventional anti-leukemia therapies due to the development of resistance and relapse (de Groot et al., 2021; Candelli et al., 2022). Interestingly, high dose MLL-AF4 protein is toxic to leukemia cells. Proteasome inhibitor (PI) bortezomib treatment selectively kills *MLL-AF4* B-ALL cells by preventing the degradation of MLL-AF4 protein (Liu et al., 2014). However, the emergence of drug tolerance has limited the clinical use of bortezomib for B-ALL treatment (Ge et al., 2020). PI-DTP cells were studied by the treatment of human *MLL-AF4* B-ALL cells with bortezomib *in vitro* culture, which display a slow-cycling and stemness feature. Ge et al. (2021b) found that PI-DTP cells display cell cycle arrest and stem cell pathway activation. Reduction of histone epigenetic markers H2BK120^ub^ and H3K4^me3^ contribute to the emergence of PI-DTP cells by downregulation of CCNA1. Inhibition of deubiquitinating enzyme by P5091 or WP 1130 can largely reverse the sensitivity of PI-DTP cells to bortezomib-treatment by restoring the expression of CCNA1 and inducing the cell cycle entry. More interestingly, such PI-tolerance of B-ALL cells can be transmitted to sensitive cells by releasing exosomes (Ge et al., 2020). Inhibition of exosome secretion by GW 4869 (a neutral sphingomyelinase inhibitor) can partially restore proliferation and sensitivity to PI-DTP cells to bortezomib treatment. Xenograft mouse model further verified that GW 4869 and bortezomib combination treatment are more effective in leukemia inhibition than bortezomib-treatment (Ge et al., 2020). These studies indicate that in ALL, the development of DTP cells is mediated by transcriptional and epigenetic machinery and regulated by unique signaling pathways.

### 3.3 DTP cells in *multiple* myeloma (MM)

MM is a neoplastic plasma cell disease characterized by clonal proliferation of malignant plasma cells (Cohen et al., 2021; Vrtis, 2024). Introduction of PIs and immunomodulators in clinical for MM treatment significantly improve patient outcomes, but relapses and resistance eventually occur in almost all patients (Ge et al., 2021a). Ge et al. (2021a) found that, upon PI treatment-induced DTP cells in MM cells are slow-cycling and display a reversible drug-tolerant state. Gene expression profiling analysis demonstrated that this reversible phenotype of PI-DTP cells is regulated by epigenetic plasticity. Histone deacetylase inhibitor and PI combination can more effectively kill MM cells by preventing the emergence of PI-tolerant cells. Translocation t (11; 14) occurs in 15%–20% of MM patients which is associated with upregulation of *CCND1* and *BCL2* (Fonseca et al., 2004; Kumar et al., 2017; Tang et al., 2025). Thus patients t (11; 14) MM are susceptibility to BCL2 inhibitor treatment (Touzeau et al., 2014). BCL2 inhibitor Venetoclax (Ven) has shown meaningful clinical activity in MM harboring the t (11:14) as either monotherapy or combination therapy. Despite the high response rates and prolonged progression-free survival, relapses are still inevitable in a significant proportion of patients (Deng et al., 2024). Deng et al. (2024), found that the reduction of mitochondrial priming, MCL1 upregulation and PUMA downregulation in Ven-induced MM drug-tolerant expanded persister (DTEP) cells are primarily mediated by epigenetic mechanism. Ven-MM DTEP cells also tolerate to standard therapeutic drugs including alkylating agents, PIs and dexamethasone, fortunately, they remain sensitive to CAR-T cell immunotherapy, suggesting CAR-T therapy might be an effective salvage therapy for Ven-resistant patients.

### 3.4 DTP cells in B *Cell* lymphoma (BCL)

BCL is a heterogeneous group of tumors of B lymphocytes (Patil et al., 2025). Current treatments for BCL include chemotherapy, radiation therapy, targeted therapy and CAR-T therapy. However, there are different degrees of relapse after the treatments (Yin et al., 2024; Patil et al., 2025). Ven is an effective treatment for some subtypes of BCL that have dysregulated BCL-2 including mantle cell lymphoma (MCL) and double-hit lymphoma (DHL), but patients who initially respond to Ven treatment develop resistance during or after treatments (Zhao et al., 2019). Zhao et al. (2019) demonstrated that Ven-resistance in MCL and DHL models is due to the selection clones with loss of *BCL2* amplificons (in cases harboring 18q21 amplications) and adaptive alteration of super-enhancer (SE) -driven transcriptional reprogramming. Ven-DTEP cells display downregulation of *BCL2* or SE-driven upregulation of other pro-survival genes *BCL2A1, BCL11A, and FOXC1.* Akin to adaptive SE-transcriptional programs-associated Ven resistance in solid tumors (Rusan et al., 2018), CDK7 inhibition can restore Ven-sensitivity in Ven-DTEP cells by repressing SE-driven expression of pro-survival genes and further reducing the expression of BCL2. Thus, a combination of CDK7 inhibitor and Ven treatment is not only able to inhibit the emergence of Ven-DTEP cells but also reduce Ven-DTEP cells.

In Table 2, we summarize the research progress on DTP cells in hematologic malignancies. Which evade apoptosis in targeted and conventional therapies and are a major nongenetic factor impeding the efficacy of hematologic tumors. Enhancing further research on DTP cells and exploring combination regimens of existing therapeutic options with targeting DTP cells to eliminate DTP cells more effectively may improve our understanding of drug resistance and relapse in hematologic tumors and enable the development of more effective antitumor therapies.

**TABLE 2:** Summary of DTP cells studies in hematological malignancies.

Cancer types	Study models	Treatments	Characteristics of DTP cells	Mechanism of DTP cells formation	Privotal acting molecules/processes	Proposed strategies for targeting DTP cells
AML (Morgenstern et al., 2025)	Molm13, MV4-11, THP1	Daunorubicin and Ara-C	1. Slowed proliferation/cycle arrest 2. Reversible tolerance to initial treatment		Fatty acid prolongation and increased sphingolipid abundance; Increases membrane rigidity	T-cell-based immunotherapy
FLT3-mutant AML (Gebru et al., 2020)	MV4-11	quizartinib	1. Cycle arrest 2. Reversible tolerance to initial treatment		Upregulation of inflammatory pathway	quizartinib + dexamethasone
TP53-mutant AML (Kim et al., 2024)	Kasumi-1, Mono-Mac-1	Cytarabine and Idarubicin	1. Cycle arrest		Elimusertib reactivates the cell cycle in TP53^mut^ DTP cells	Cytarabine, idarubicin, and elimusertib
AML (van Gils et al., 2022)	K562	doxorubicin	1.Transient tolerance to Reversible tolerance to initial treatment 2. Stem cell phenotype	Transcriptional reprogramming	H3K27me3, H3K4me3, KDM6	GSK-J4+ doxorubicin
T-ALL (Knoechel et al., 2014)	DND-41 KOPT-K1	GS1	1. Growth arrest 2. Reversible tolerance to initial treatment	Epigenetic mechanism	BRD4	GSI + JQ1
ALL (Malyukova et al., 2024)	RS4; 11	AZD1775	1. Slowed proliferation/cycle arrest, 2. Reversible tolerance to initial treatment	Epigenetic reprogram		Novel sequential drug administration (AZD1775 + dasatinib or ibrutinib or fatostatin or AZD2014)
Pro-B-ALL harboring MLL-AF4 (Ge et al., 2020)	RS4; 11; SEM	Bortezomib	1. Reversible tolerance to initial treatment 2. Cycle arrest; 3. Stem cell-like phenotype	Non–autonomous mechanisms		Bortezomib + GW4869
Pro-B-ALL harboring MLL-AF4 (Ge et al., 2021b)	RS4; 11 and SEM	Bortezomib	Reversible tolerance to initial treatment	Epigenetic reprogramming	CCNA1	Bortezomib + deubiquitinating enzyme (DUB) inhibitors
MM (Ge et al., 2021a)	MM1.S and RPMI-8226	Bortezomib	1. Reversible tolerance to initial treatment 2. Cycle arrest/Slowed proliferation	Epigenetic alterations	Epigenetic regulators	PIs + HDAC inhibitors
MM (Deng et al., 2024)	KMS12PE KMS27	venetoclax	Reversible tolerance to initial treatment		Reduced mitochondrial priming and changes in BCL-2 family protein expression	Immunotherapeutic strategies
BCL (Zhao et al., 2019)	HBL-2	venetoclax	Reversible tolerance to initial treatment	Loss of *BCL2* amplificons; transcriptional reprogramming	BCL2, BCL2A1, BCL11A, and FOXC1	THZ1+venetoclax

GSK-J4, KDM6 inhibitor; GS1, γ-secretase inhibitors; JQ1, BRD4 inhibitor; AZD1775, WEE1 inhibitor; GW4869, neutral sphingomyelinase inhibitor; PIs, Proteasome inhibitors; THZ1, CDK7 inhibitor.

## 4 Possible strategies for targeting DTP cells for treatment of hematologic malignancies

**FIGURE 3:**
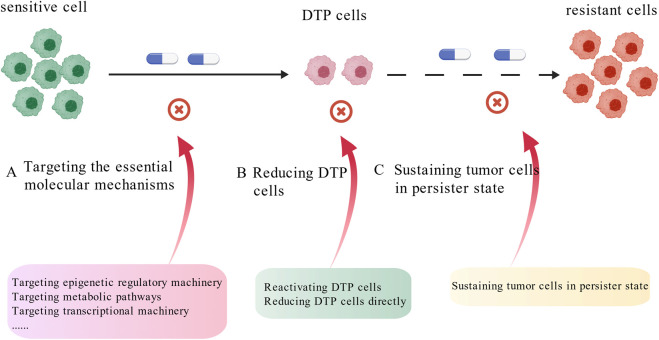
Targeted DTP cells Strategy. Three distinct strategies aimed to **(A)** Targeting the essential molecular mechanisms, **(B)** Reducing DTP cells, **(C)** Sustaining tumor cells in persister state (Created with BioGDP.com).

Due to the drug- and cancer type-specific mechanisms of DTP cells development, targeting DTP cells therapies should be different among cancer types in a primary treatment-dependent manner. Thus, to target DTP cells for long-lasting treatment effects of cancers, it is very important to elucidate the distinct molecular mechanism of DTP cells development in each type of cancer in response to specific drug treatment. Three strategies have been proposed to target DTP cells. The first strategy is to inhibit the essential mechanism of DTP cells development together with primary drug simultaneously to prevent DTP cells generation. The second strategy is, immediately after standard treatment, targeting the vulnerable molecules for DTP cells survival to reduce DTP cells. The third strategy is to inhibit the key machinery for DTP cells reactivation at the time of disease remission to maintain DTP cells in a dormant persister state and prevent disease relapse (Figure 3).

### 4.1 Targeting the essential molecular mechanisms to prevent the generation of DTP cells

The reversible drug resistance in DTP cells is primarily mediated by drug-induced epigenetic landscape changes which alternate transcriptional profile, driving a biologic and metabolic fitness of the cells to drug toxic environment. Therefore, DTP cells can be targeted by inhibiting the key epigenetic modifiers, transcriptional machinery and/or metabolic regulators that are essential for the establishment of DTP cells.

#### 4.1.1 Targeting epigenetic regulatory machinery

Histone remodifications including methylation, acetylation and ubiquitination of histone tails are the most common type of epigenetic alterations detected in DTP cells from hematopoietic malignancies due to the changes in histone methylation and acetylation modifiers (Chen, 2019; He et al., 2024). H3K4 tri-methylation (H3K4^me3^) and H3K27 ^me3^ marker active and repressive gene transcriptions respectively in cells. H3K4^me3^ is written by MLL family of lysine methyltransferases and is removed by H3K4 demethylase KDM5 (Wang and Helin, 2025). H3K27 ^me3^ is catalyzed by EZH2, a component of the PRC2 complex, and is removed by H3K27 demethylase KDM6. Chemotherapy drug doxorubicin induces both H3K27^me3^ and H3K4^me3^ at the promotors of target genes which are associated with AML cell killing. Doxorubicin-induced AML DTP cells are marked by reduced H3K27^me3^, most likely due to the enhanced KDM6B and reduced EZH1 and EZH2 expression. Consequently, genetic or pharmacologic inhibition of KDM6B facilitates the elimination of DTP cells and enhances the anti-AML effect of doxorubicin *in vivo* (van Gils et al., 2022). Consistently, in clinical studies, loss of EZH2, upregulation of KDM6 and reduced H3K27^Me3^ are associated with multiple drug resistance and poor prognosis of AML patients (Gollner et al., 2017; Li et al., 2018; Maganti et al., 2018). Active enhancers are marked by H3K4^Me1/2^ which is catalyzed by MLL3/4 and removed by LSD1 (KDM1) and LSD2 (KDM2). IBET-151 (a novel selective BET inhibitor for BRD2, BRD3 and BRD4) treatment kills AML cells by repressing mediator complex-modulated enhancer-promotor interaction. IBET-151-AML DTP cells are characterized by reduced access of Pu1 and Irf8 to the enhancers of target genes due to Lsd1 upregulation. Lsd1 inhibition reactivates the enhancers for Pu.1 and Irf8 binding, resensitizing DTP cells to IBET-151 treatment (Bell et al., 2019). Both H3K4^Ac^ and H3K27^Ac^ are catalyzed by Histone acetyltransferases (HATs) and erased by Histone deacetylases (HDACs), marking active genes. PI-DTP cells from MM patients display significant enrichment of HDAC genes. HDAC inhibitors panobinostat (LBH589) and vorinostat (SAHA) resensitize DTP cells to PI treatment, suggesting a potential of combination of HDAC inhibitor plus PI for MM treatment (Ge et al., 2021a).

#### 4.1.2 Targeting metabolic pathways

Metabolic reprogramming is one of the most extensively studied features of DTP cells, and its targeting has been widely explored in solid tumors (Hangauer et al., 2017; Shen et al., 2020; Oren et al., 2021). In the early phase of MRD following chemotherapy in AML patients, cachexia-like metabolic reprogramming may support the energy demands for leukemia regeneration and relapse. Farge et al. (2017) demonstrated that targeting lipid-oxidation and mitochondrial metabolism by targeting CD36-FAO-OXPHOS axis induces a shift toward low OXPHOS and significantly enhances the anti-leukemic effects of AraC. This study provides theoretical support for targeting mitochondrial reprogramming to prevent tumor recurrence.

#### 4.1.3 Targeting transcriptional machinery

An example of targeting transcriptional reprogramming involves Ven-induced DTEP cells in MCL cells. Transcriptional CDK7, a transcriptional CDK, plays essential role in regulating transcription initiation and elongation by phosphorylating key serine residues of the C-terminal domain of RNAPII. Ven-MCL DTEP cells are cell cycle quiescent, which expansion and survival are driven by CDK7-dependent SE-regulated transcriptional reprogramming. Consequently, combination of a CDK7 inhibitor (THZ1) and Ven not only prevents the emergence of Ven-DTEP cells but also eliminates Ven-DTEP cells after their development (Zhao et al., 2019).

### 4.2 Reducing DTP cells

#### 4.2.1 Reactivating DTP cells

Slow cell cycle is a common feature of DTP cells which is one of the mechanisms for DTP cells resistance, because most current anti-cancer drugs specifically chemotherapeutic drugs selectively kill proliferative cells. Thus, inducing DTP cells to a proliferative state might enhance the efficacy of treatment (Recasens and Munoz, 2019; Mikubo et al., 2021). In AML, G-CSF stimulation can induce quiescent LSC-like DTP cells to enter the active cell cycle, thereby enhancing the efficacy of chemotherapy (Saito et al., 2010; Di Tullio et al., 2017). In PI treated MLL-AF4 ALL cells, DTP cells are arrested in G0/G1 phases due to reduction of H2BK120^ub^ and its associated downregulated Cyclin A1 (Ge et al., 2021b). H2BK120^Ub^ is catalyzed primarily by heteromeric RNF20/40 complex (Kim et al., 2005; Zhu et al., 2005; Duzanic et al., 2025) and deubiquitinated by several deubiquitinating enzymes (DUB) including several ubiquitin-specific proteases (as USP12, USP22, USP44 and USP46 (Zhang et al., 2008; Fuchs et al., 2012; Joo et al., 2011). DUB inhibitors increased H2B120^ub^ -dependent H3K4 methylation and induced Cyclin A1 expression, providing a theoretical basis for combining DUB inhibitor plus PI for MLL-AF4 ALL treatment (Ge et al., 2021b). However, in AML harboring TP53 mutations, DTP cells are arrested in G2/M phases of cell cycle, elimusertib inhibits the expression of the G2/M-phase cell-cycle checkpoints regulators (including cdc25c, p-CDK1, and Cyclin b) to reactivate the cell cycle thereby resensitize to treatments (Kim et al., 2024). All of the above studies indicate that re-entering the cell cycle may serve as a therapeutic strategy targeting DTP cells. However, caution is warranted regarding the potential for reactivated tumor cells to acquire increased proliferation/invasiveness or drug resistance (Mikubo et al., 2021). Thus, if the awakening of DTP cells is not followed by highly efficacious anti-proliferative agents, this strategy could significantly worsen the patient’s prognosis (Recasens and Munoz, 2019).

#### 4.2.2 Reducing DTP cells directly

When gain tolerate to certain treatment, the DTP cells might simultaneously obtain some other epigenetic/metabolic weaknesses, thus targeting these weaknesses (vulnerabilities) might provide safe and specific mechanisms to reduce DTP cells. This method has been thoroughly validated in solid tumors. For instance, DTP cells derived from breast, melanoma, lung, and ovarian cancer cells can be cleared by ferroptosis activators RSL3 and ML210 through the inhibition of glutathione peroxidase 4 (GPX4) (Yang et al., 2014; Hangauer et al., 2017; Recasens and Munoz, 2019). The findings in solid tumors have greatly encouraged us to delve deeper into exploring DTP cells that directly target the origin of hematologic malignancies. Existing research indicates that AML-derived DTP cells exhibit transient increases in cytoplasmic membrane rigidity following induction of cytarabine and doxorubicin, conferring therapeutic resistance and simultaneously increasing neoantigen presentation. Employing T-cell immunotherapy after induction chemotherapy may serve as an effective approach to reduce such DTP cells (Morgenstern et al., 2025). The elevated inflammatory-survival signaling in FLT3-induced AML-derived DTP cells provides an opportunity to reduce them by combined treatment with FLT3 inhibitor and glucocorticoid (Gebru et al., 2020).

### 4.3 Sustaining tumor cells in persister state

DTP cells derived from most drug treated cancers are maintained in a dormancy-like state (temporary cell cycle arrest), which need to be activated and reenter to cell cycle for expansion and tumor relapse, thus inhibiting the cell cycle reentry of DTP cells has been proposed as a novel strategy to prevent disease relapse. To do so, it is very important to leverage the factors and signaling pathways that are essential for cell cycle reentry of DTP cells (Recasens and Munoz, 2019).

Although CDK4/6 are the master regulators for G0/G1 phase to S phase cell cycle transition (Finn et al., 2015; Bollard et al., 2017; Recasens and Munoz, 2019). Oral administration of CDK4/6 inhibitor palbociclib can induce prolonged G1 cell cycle arrest. Martin et al. (2019). Found that palbociclib synergistically killed Bruton’s tyrosine kinase (BTK) inhibitor ibrutinib-resistant MCL cells most likely through inhibition of compensatory signaling pathways, principally PI3k (Chiron et al., 2014). A Phase 1 trial demonstrated the dose, safety, preliminary activity and objective efficacy of palbociclib in combination with ibrutinib in previously treated MCL patients (Martin et al., 2019). CDK9 forms a superenhancer complex with cyclin T1, positive transcription elongation factor to regulate the activity of RNA-polymerase II, playing critical role in regulate the levels of the faster turnover proteins such as MCL1 and cMYC (Lee and Zeidner, 2019). In newly diagnosed and relapsed/refractory AML patients, CDK9 inhibitor Alvocidib inhibits MCL1 and cMYC expression enhanced the treatment efficacy of cytarabine and mitoxantrone in newly diagnosed and relapsed/refractory AML patients (Lee and Zeidner, 2019). The above-mentioned research suggests that blocking the re-entry of residual hematological tumor cells after drug treatment into the cell cycle can achieve deeper remission and thus serve as a new strategy for preventing disease recurrence. Therefore, how to sustain persistence and inhibit proliferative signaling to keep tumor cells in this “dormant” state permanently will be a challenge for future research.

## 5 Challenges and future research directions for DTP cells

The mechanistic studies of DTP cells in hematological malignancies are still in an infant state, therefore targeting DTP cells to prevent or reduce drug resistance and disease relapse, remains both a challenge and an opportunity.

1. Although comprehensive clinical data specifically targeting DTP cells therapy remain limited, existing evidence demonstrates that that maintenance chemotherapy following induction therapy may be related to this concept (Mikubo et al., 2021). Adjuvant chemotherapy following solid tumor surgery and maintenance therapy for hematologic malignancies effectively improve patient prognosis while reducing the likelihood of cancer recurrence and metastasis. This suggests an alternative perspective for anti-DTP therapy: transitioning to long-term maintenance treatment following existing combination anticancer regimens. This shift would redirect focus from treating MRD to targeting its potential origin—DTP cells. For instance, maintenance therapy with venetoclax or azacitidine has shown favorable outcomes for AML patients (Kungwankiattichai et al., 2022; Bazinet et al., 2024). Unfortunately, there is currently no standardized regimen regarding the duration and dosage of maintenance treatment (Ali et al., 2020). The application of the DTP cells concept may help resolve this controversy.
2. Currently, there is a lack of highly specific biomarkers for identifying DTP cells, making it impossible to accurately identify, isolate, and dynamically monitor this cell population in patients. In the future, it is anticipated that potential DTP cells biomarkers may be explored through single-cell RNA sequencing (scRNA-seq) (Xue et al., 2025). Liquid biopsy could enable real-time, dynamic monitoring of DTP cells during treatment, thereby providing precise decision-making support for targeted DTP cells (Wang R. et al., 2025). Therefore, developing more precise technologies and identifying specific biomarkers will be key challenges for the future.
3. DTP cells frequently induces cross-drug resistance. For example, MLL DTP cells resistant to bortezomib also exhibit resistance to carfilzomib (Ge et al., 2020). Venetoclax-resistant multiple myeloma cell lines exhibit broad resistance to standard anti-myeloma drugs (Deng et al., 2024). Cross-resistance to daunorubicin was similarly demonstrated in a model of doxorubicin-induced AML DTP cells (van Gils et al., 2022). This represents a detrimental phenomenon observed in patients with poor response to subsequent therapies. Therefore, developing strategies to better leverage DTP cells for targeting multidrug resistance represents a critically important direction for future research.
4. Optimizing drug administration regimens, determining the optimal timing and sequence of treatments, managing potential adverse reactions, and ultimately designing rational clinical trials for translation represent an area that requires further exploration. Historically, it was believed that maximizing drug doses and prolonging treatment duration could effectively kill tumor cells and prevent the development of drug resistance. However, a recent clinical study reported that shorter courses (7 or 14 days) of Ven in ND-AML may be equally effective as the standard 28-day regimen while causing less bone marrow toxicity (Gangat and Tefferi, 2025). This phenomenon appears to be explained by DTP cells. Furthermore, based on current research on DTP cells, anti-DTP combined with anti-tumor therapy represents a promising approach. However, while DTP cells offer opportunities for re-sensitization, they also imply potentially unstable states, making the therapeutic window difficult to grasp. Identifying the optimal timing for targeting DTP cells remains a challenge for future research. For instance, in FLT3-mutated AML DTP cells, quazatinib-induced DTP cells emerged within 48 h of dosing. This finding suggests clinicians may target DTP cells as early as 48 h after initiating quazatinib treatment (Gebru et al., 2020). Additionally, beyond combination therapy, intermittent dosing strategies have also demonstrated therapeutic potential. In MM, PI combination therapy with anti-DTP and high-dose intermittent treatment have been found to prevent the emergence of PI-tolerant cells, thereby more effectively treating MM (Ge et al., 2021a). Of course, drug interactions and adverse reactions caused by drug combinations require further investigation. Therefore, designing large-scale clinical trials to further validate that targeting DTP cells delays drug resistance development and tumor recurrence will be the next major focus in overcoming tumor resistance.

## 6 Conclusion

As key factors in tumor drug resistance and recurrence, DTP cells play a significant role in hematologic malignancies. It achieve tolerance to anticancer drugs through mechanisms such as epigenetic modifications, transcriptional regulation, and metabolic reprogramming. Several targeted strategies have been developed to exploit the specific weaknesses of DTP cells. However, DTP cells also face challenges, such as the lack of clinical biomarkers or validated DTP cells-targeted therapies. As the understanding of the biological characteristics of DTP cells deepens and technological methods continue to advance, it is anticipated that in the near future, we will be able to identify their key clinical biomarkers and develop innovative therapies capable of delaying relapse and overcoming drug resistance. This will bring new hope for achieving long-term remission and even a cure for hematologic malignancies.
